# 2,3-Dimethyl-*N*-[(*E*)-4-nitro­benzyl­idene]aniline

**DOI:** 10.1107/S160053681001932X

**Published:** 2010-06-05

**Authors:** Muhammad Ilyas Tariq, Shahbaz Ahmad, M. Nawaz Tahir, Muhammad Sarfaraz, Ishtiaq Hussain

**Affiliations:** a Department of Chemistry, University of Sargodha, Sargodha, Pakistan; b Department of Physics, University of Sargodha, Sargodha, Pakistan

## Abstract

In the title compound, C_15_H_14_N_2_O_2_, the aromatic rings are oriented at a dihedral angle of 24.52 (5)°. The dihedral angle between the nitro group and its parent benzene ring is 9.22 (16)°. In the crystal, mol­ecules inter­act through aromatic π—π stacking inter­actions [centroid–centroid separations = 3.8158 (14) and 3.9139 (14) Å].

## Related literature

For structural systematics of related compounds, see: Harada *et al.* (2004 ▶).
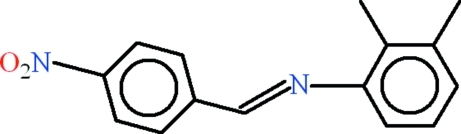


## Experimental

### 

#### Crystal data

C_15_H_14_N_2_O_2_
*M* *_r_* = 254.28
Orthorhombic, 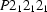

*a* = 7.1969 (5) Å
*b* = 11.8023 (7) Å
*c* = 15.3721 (8) Å
*V* = 1305.71 (14) Å^3^
*Z* = 4
Mo *K*α radiation
μ = 0.09 mm^−1^
*T* = 296 K
0.32 × 0.14 × 0.14 mm

#### Data collection

Bruker Kappa APEXII CCD diffractometer
Absorption correction: multi-scan (*SADABS*; Bruker, 2005 ▶) *T*_min_ = 0.986, *T*_max_ = 0.987
13172 measured reflections
1917 independent reflections
1253 reflections with *I* > 2σ(*I*)
*R*_int_ = 0.057

#### Refinement

*R*[*F* ^2^ > 2σ(*F* ^2^)] = 0.047
*wR*(*F* ^2^) = 0.111
*S* = 1.02
1917 reflections
174 parameters
H-atom parameters constrained
Δρ_max_ = 0.12 e Å^−3^
Δρ_min_ = −0.14 e Å^−3^

### 

Data collection: *APEX2* (Bruker, 2007 ▶); cell refinement: *SAINT* (Bruker, 2007 ▶); data reduction: *SAINT*; program(s) used to solve structure: *SHELXS97* (Sheldrick, 2008 ▶); program(s) used to refine structure: *SHELXL97* (Sheldrick, 2008 ▶); molecular graphics: *ORTEP-3 for Windows* (Farrugia, 1997 ▶) and *PLATON* (Spek, 2009 ▶); software used to prepare material for publication: *WinGX* (Farrugia, 1999 ▶) and *PLATON*.

## Supplementary Material

Crystal structure: contains datablocks global, I. DOI: 10.1107/S160053681001932X/hb5464sup1.cif

Structure factors: contains datablocks I. DOI: 10.1107/S160053681001932X/hb5464Isup2.hkl

Additional supplementary materials:  crystallographic information; 3D view; checkCIF report

## Figures and Tables

**Fig. 1.**
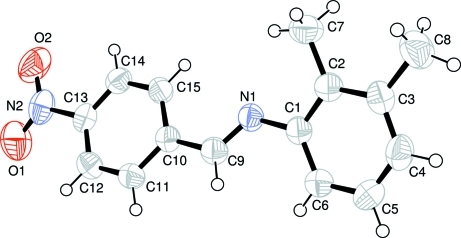
View of (I) with displacement ellipsoids drawn at the 50% probability level.

## supplementary crystallographic information

### Comment

Torsional, vibration and central bond length of *N*-benzylideneanilines (Harada *et al.*, 2004) has been studied for seven compounds at different temperatures. The title compound (I, Fig. 1) is another example due to change of substitutions at both phenyl rings for which the same study can be undertaken. The title compound has been prepared for derivatization.

The molecules of (I) are essentially monomeric having no intra or inter-molecular H-bondings. The phenyl rings A (C1—C6) and B (C10—C15) are planar with r. m. s. deviation of 0.0065 and 0.0022 Å respectively. The dihedral angle between A/B is 24.52 (5)°. The nitro group C (O1/N2/O2) is oriented at 9.22 (16)° with the parent phenyl ring. It is to be noted that the nitro substituated phenyl ring B has smaller bond lengths [1.365 (3)–1.387 (3) Å], whereas the 2,3-dimethyl substituated ring has longer bond lengths 1.373 (3)—1.401 (3) Å. The value of C═N bond length at room temperature for (I) is 1.262 (3) Å which is in compatible with the studies of Harada *et al.*, 2004. The molecules are stabilized due to π—π interactions between the centroids of phenyl rings with separation of 3.8158 (14) and 3.9139 (14) Å.

### Experimental

Equimolar quantities of 2,3-dimethylaniline and 4-nitro benzaldehyde were refluxed in methanol for 15 minutes resulting in yellow color precipitates. The contents of the flask were dried at room temperature and washed with methanol and  ethanol, respectively. The washed crude material affoarded yellow needles of (I) in the solution of diethyl ether at room temperature by slow evaporation after 24 h.

### Refinement

In the absence of anomalous scattering, all Friedal pairs were merged. Although all H-atoms were appeared in Fourier difference map but positioned geometrically (C–H = 0.93–0.96 Å) and refined as riding with *U*_iso_(H) = *xU*_eq_(C), where *x* =1.2 for aryl C–H and *x* = 1.5 methyl H-atoms.

### Crystal data

**Table:**

C_15_H_14_N_2_O_2_	*F*(000) = 536
*M**_r_* = 254.28	*D*_x_ = 1.294 Mg m^−^^3^
Orthorhombic, *P*2_1_2_1_2_1_	Mo *K*α radiation, λ = 0.71073 Å
Hall symbol: P 2ac 2ab	Cell parameters from 1334 reflections
*a* = 7.1969 (5) Å	θ = 2.1–25.4°
*b* = 11.8023 (7) Å	µ = 0.09 mm^−^^1^
*c* = 15.3721 (8) Å	*T* = 296 K
*V* = 1305.71 (14) Å^3^	Needle, yellow
*Z* = 4	0.32 × 0.14 × 0.14 mm

### Data collection

**Table:**

Bruker Kappa APEXII CCD diffractometer	1917 independent reflections
Radiation source: fine-focus sealed tube	1253 reflections with *I* > 2σ(*I*)
graphite	*R*_int_ = 0.057
Detector resolution: 7.40 pixels mm^-1^	θ_max_ = 28.6°, θ_min_ = 2.2°
ω scans	*h* = −8→9
Absorption correction: multi-scan (*SADABS*; Bruker, 2005)	*k* = −15→15
*T*_min_ = 0.986, *T*_max_ = 0.987	*l* = −20→20
13172 measured reflections	

### Refinement

**Table:**

Refinement on *F*^2^	Primary atom site location: structure-invariant direct methods
Least-squares matrix: full	Secondary atom site location: difference Fourier map
*R*[*F*^2^ > 2σ(*F*^2^)] = 0.047	Hydrogen site location: inferred from neighbouring sites
*wR*(*F*^2^) = 0.111	H-atom parameters constrained
*S* = 1.02	*w* = 1/[σ^2^(*F*_o_^2^) + (0.0482*P*)^2^ + 0.0883*P*] where *P* = (*F*_o_^2^ + 2*F*_c_^2^)/3
1917 reflections	(Δ/σ)_max_ < 0.001
174 parameters	Δρ_max_ = 0.12 e Å^−^^3^
0 restraints	Δρ_min_ = −0.14 e Å^−^^3^

### Special details

**Table:**

Geometry. Bond distances, angles *etc*. have been calculated using the rounded fractional coordinates. All su's are estimated from the variances of the (full) variance-covariance matrix. The cell e.s.d.'s are taken into account in the estimation of distances, angles and torsion angles
Refinement. Refinement of *F*^2^ against ALL reflections. The weighted *R*-factor *wR* and goodness of fit *S* are based on *F*^2^, conventional *R*-factors *R* are based on *F*, with *F* set to zero for negative *F*^2^. The threshold expression of *F*^2^ > σ(*F*^2^) is used only for calculating *R*-factors(gt) *etc*. and is not relevant to the choice of reflections for refinement. *R*-factors based on *F*^2^ are statistically about twice as large as those based on *F*, and *R*- factors based on ALL data will be even larger.

### Fractional atomic coordinates and isotropic or equivalent isotropic displacement parameters (Å^2^)

**Table:**

	*x*	*y*	*z*	*U*_iso_*/*U*_eq_	
O1	0.1409 (4)	−0.19301 (18)	0.70198 (14)	0.0997 (9)	
O2	0.1083 (4)	−0.06237 (19)	0.79842 (12)	0.0941 (9)	
N1	0.1128 (3)	0.34130 (16)	0.47092 (11)	0.0485 (7)	
N2	0.1253 (3)	−0.0943 (2)	0.72311 (16)	0.0684 (9)	
C1	0.1231 (3)	0.42218 (18)	0.40278 (13)	0.0457 (7)	
C2	0.1509 (3)	0.5351 (2)	0.42653 (14)	0.0488 (8)	
C3	0.1581 (4)	0.61833 (19)	0.36160 (15)	0.0549 (8)	
C4	0.1353 (4)	0.5864 (2)	0.27595 (17)	0.0624 (10)	
C5	0.1052 (4)	0.4755 (2)	0.25272 (16)	0.0650 (10)	
C6	0.0972 (4)	0.3935 (2)	0.31593 (14)	0.0550 (8)	
C7	0.1768 (4)	0.5654 (2)	0.52133 (14)	0.0679 (10)	
C8	0.1878 (5)	0.7409 (2)	0.38401 (19)	0.0820 (13)	
C9	0.1573 (4)	0.23940 (19)	0.45751 (14)	0.0495 (8)	
C10	0.1434 (3)	0.15342 (18)	0.52625 (14)	0.0448 (7)	
C11	0.1832 (3)	0.04156 (19)	0.50760 (15)	0.0525 (8)	
C12	0.1753 (4)	−0.0397 (2)	0.57139 (15)	0.0551 (9)	
C13	0.1287 (3)	−0.0075 (2)	0.65402 (14)	0.0500 (8)	
C14	0.0878 (3)	0.1021 (2)	0.67504 (15)	0.0523 (8)	
C15	0.0953 (3)	0.18289 (19)	0.61064 (14)	0.0496 (8)	
H4	0.14039	0.64145	0.23274	0.0748*	
H5	0.09027	0.45617	0.19450	0.0779*	
H6	0.07448	0.31854	0.30059	0.0659*	
H7A	0.09112	0.62429	0.53696	0.1016*	
H7B	0.30167	0.59128	0.53059	0.1016*	
H7C	0.15399	0.49972	0.55663	0.1016*	
H8A	0.18433	0.78558	0.33184	0.1229*	
H8B	0.30655	0.74981	0.41166	0.1229*	
H8C	0.09161	0.76571	0.42285	0.1229*	
H9	0.20007	0.21822	0.40278	0.0594*	
H11	0.21572	0.02105	0.45120	0.0630*	
H12	0.20116	−0.11509	0.55867	0.0661*	
H14	0.05543	0.12165	0.73165	0.0627*	
H15	0.06802	0.25797	0.62382	0.0596*	

### Atomic displacement parameters (Å^2^)

**Table:**

	*U*^11^	*U*^22^	*U*^33^	*U*^12^	*U*^13^	*U*^23^
O1	0.131 (2)	0.0586 (12)	0.1094 (16)	0.0005 (15)	0.0004 (17)	0.0307 (12)
O2	0.1205 (19)	0.1055 (16)	0.0563 (11)	−0.0025 (15)	−0.0044 (13)	0.0275 (12)
N1	0.0509 (12)	0.0484 (11)	0.0463 (11)	0.0052 (10)	0.0029 (10)	0.0042 (9)
N2	0.0614 (15)	0.0707 (16)	0.0731 (16)	−0.0068 (14)	−0.0072 (13)	0.0266 (13)
C1	0.0431 (14)	0.0477 (12)	0.0463 (12)	0.0035 (11)	0.0067 (11)	0.0043 (10)
C2	0.0454 (15)	0.0529 (13)	0.0482 (12)	0.0020 (12)	0.0069 (11)	0.0015 (11)
C3	0.0515 (16)	0.0504 (13)	0.0628 (15)	0.0051 (12)	0.0070 (12)	0.0082 (11)
C4	0.0660 (19)	0.0615 (16)	0.0596 (16)	0.0045 (15)	0.0068 (14)	0.0193 (13)
C5	0.079 (2)	0.0708 (17)	0.0453 (12)	0.0029 (16)	0.0008 (14)	0.0040 (12)
C6	0.0614 (16)	0.0526 (14)	0.0509 (14)	0.0027 (13)	0.0009 (13)	−0.0010 (12)
C7	0.081 (2)	0.0651 (16)	0.0577 (14)	−0.0020 (16)	0.0041 (14)	−0.0097 (13)
C8	0.105 (3)	0.0539 (15)	0.087 (2)	−0.0044 (18)	0.009 (2)	0.0079 (15)
C9	0.0467 (15)	0.0539 (13)	0.0479 (12)	0.0039 (12)	0.0081 (12)	0.0010 (11)
C10	0.0406 (13)	0.0467 (12)	0.0471 (13)	0.0009 (11)	0.0030 (11)	0.0026 (10)
C11	0.0578 (16)	0.0514 (13)	0.0483 (12)	0.0057 (12)	0.0043 (11)	−0.0031 (11)
C12	0.0608 (17)	0.0424 (12)	0.0621 (15)	0.0015 (12)	0.0011 (13)	0.0007 (11)
C13	0.0436 (14)	0.0550 (14)	0.0515 (12)	−0.0029 (12)	−0.0050 (12)	0.0116 (11)
C14	0.0521 (15)	0.0601 (16)	0.0447 (12)	0.0018 (13)	−0.0007 (11)	0.0014 (12)
C15	0.0521 (15)	0.0456 (12)	0.0512 (13)	0.0046 (12)	0.0015 (12)	0.0005 (11)

### Geometric parameters (Å, °)

**Table:**

O1—N2	1.215 (3)	C12—C13	1.368 (3)
O2—N2	1.224 (3)	C13—C14	1.365 (3)
N1—C1	1.419 (3)	C14—C15	1.376 (3)
N1—C9	1.262 (3)	C4—H4	0.9300
N2—C13	1.476 (3)	C5—H5	0.9300
C1—C2	1.396 (3)	C6—H6	0.9300
C1—C6	1.390 (3)	C7—H7A	0.9600
C2—C3	1.401 (3)	C7—H7B	0.9600
C2—C7	1.512 (3)	C7—H7C	0.9600
C3—C4	1.379 (3)	C8—H8A	0.9600
C3—C8	1.502 (3)	C8—H8B	0.9600
C4—C5	1.374 (3)	C8—H8C	0.9600
C5—C6	1.373 (3)	C9—H9	0.9300
C9—C10	1.468 (3)	C11—H11	0.9300
C10—C11	1.381 (3)	C12—H12	0.9300
C10—C15	1.387 (3)	C14—H14	0.9300
C11—C12	1.373 (3)	C15—H15	0.9300
			
C1—N1—C9	120.49 (18)	C5—C4—H4	119.00
O1—N2—O2	123.9 (2)	C4—C5—H5	120.00
O1—N2—C13	118.2 (2)	C6—C5—H5	120.00
O2—N2—C13	118.0 (2)	C1—C6—H6	120.00
N1—C1—C2	117.16 (18)	C5—C6—H6	120.00
N1—C1—C6	122.56 (19)	C2—C7—H7A	109.00
C2—C1—C6	120.2 (2)	C2—C7—H7B	109.00
C1—C2—C3	119.2 (2)	C2—C7—H7C	109.00
C1—C2—C7	119.7 (2)	H7A—C7—H7B	110.00
C3—C2—C7	121.1 (2)	H7A—C7—H7C	109.00
C2—C3—C4	118.9 (2)	H7B—C7—H7C	109.00
C2—C3—C8	121.1 (2)	C3—C8—H8A	109.00
C4—C3—C8	119.9 (2)	C3—C8—H8B	109.00
C3—C4—C5	121.8 (2)	C3—C8—H8C	109.00
C4—C5—C6	119.6 (2)	H8A—C8—H8B	109.00
C1—C6—C5	120.2 (2)	H8A—C8—H8C	109.00
N1—C9—C10	121.6 (2)	H8B—C8—H8C	109.00
C9—C10—C11	119.8 (2)	N1—C9—H9	119.00
C9—C10—C15	121.1 (2)	C10—C9—H9	119.00
C11—C10—C15	119.1 (2)	C10—C11—H11	120.00
C10—C11—C12	120.7 (2)	C12—C11—H11	120.00
C11—C12—C13	118.7 (2)	C11—C12—H12	121.00
N2—C13—C12	118.7 (2)	C13—C12—H12	121.00
N2—C13—C14	118.9 (2)	C13—C14—H14	121.00
C12—C13—C14	122.4 (2)	C15—C14—H14	121.00
C13—C14—C15	118.6 (2)	C10—C15—H15	120.00
C10—C15—C14	120.6 (2)	C14—C15—H15	120.00
C3—C4—H4	119.00		
			
C9—N1—C1—C2	−153.3 (2)	C2—C3—C4—C5	0.2 (4)
C9—N1—C1—C6	30.1 (4)	C8—C3—C4—C5	−179.1 (3)
C1—N1—C9—C10	−178.4 (2)	C3—C4—C5—C6	0.1 (4)
O1—N2—C13—C12	−9.2 (3)	C4—C5—C6—C1	−1.3 (4)
O1—N2—C13—C14	171.7 (2)	N1—C9—C10—C11	176.3 (2)
O2—N2—C13—C12	170.3 (3)	N1—C9—C10—C15	−5.4 (4)
O2—N2—C13—C14	−8.8 (3)	C9—C10—C11—C12	178.5 (2)
N1—C1—C2—C3	−178.7 (2)	C15—C10—C11—C12	0.1 (3)
N1—C1—C2—C7	2.6 (3)	C9—C10—C15—C14	−178.2 (2)
C6—C1—C2—C3	−2.0 (3)	C11—C10—C15—C14	0.2 (3)
C6—C1—C2—C7	179.3 (2)	C10—C11—C12—C13	−0.5 (4)
N1—C1—C6—C5	178.8 (2)	C11—C12—C13—N2	−178.3 (2)
C2—C1—C6—C5	2.3 (4)	C11—C12—C13—C14	0.8 (4)
C1—C2—C3—C4	0.8 (4)	N2—C13—C14—C15	178.6 (2)
C1—C2—C3—C8	−180.0 (3)	C12—C13—C14—C15	−0.5 (3)
C7—C2—C3—C4	179.4 (2)	C13—C14—C15—C10	0.0 (3)
C7—C2—C3—C8	−1.3 (4)		
